# Supplementary material for: Cytokines and Pancreatic Ductal Adenocarcinoma: Exploring Their Relationship with Molecular Subtypes and Prognosis
Source: Int J Mol Sci. 2024 Aug 29;25(17):9368. doi: 10.3390/ijms25179368 (PMC11395259; doi:10.3390/ijms25179368)
Supplement: Supplementary file 1 [file ijms-25-09368-s001.zip › ijms-3141133-supplementary.pdf]

Supplementary Figure S1: Flowchart illustrating the selection and inclusion of patients in the study.

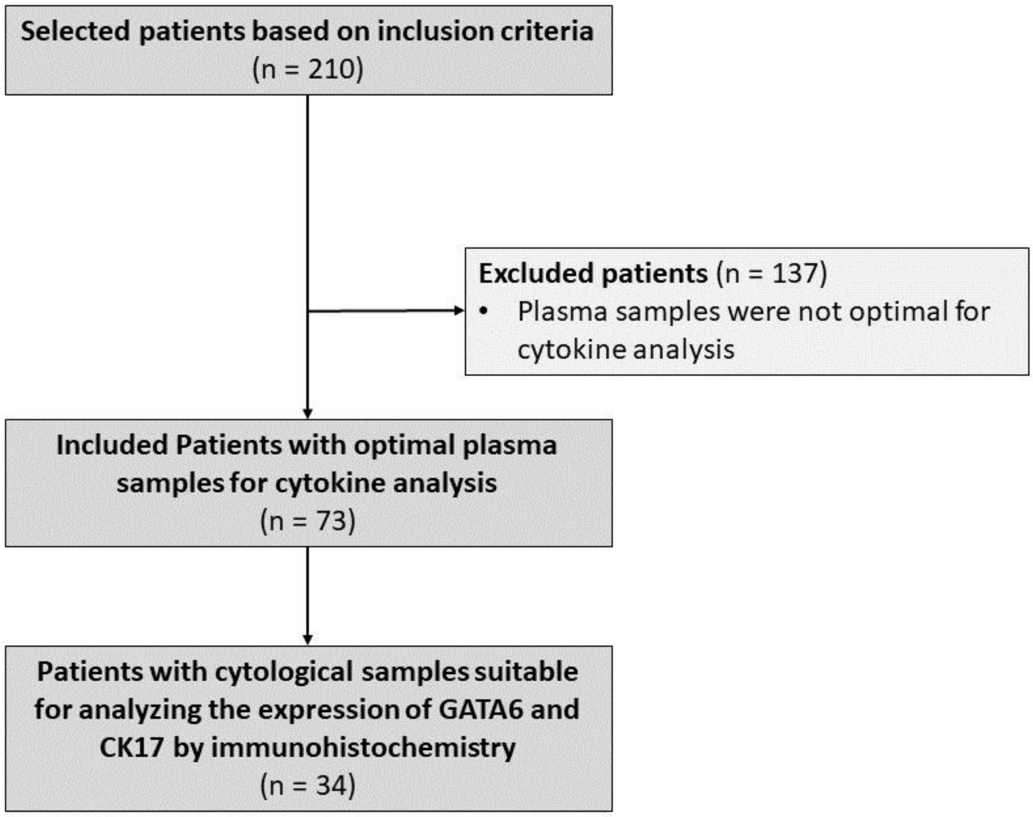

Supplementary Figure S2: Kaplan-Meier curves for progression-free survival (PFS) during first-line treatment (A) and overall survival (OS) (B) in patients with classical versus basal-like subtypes.

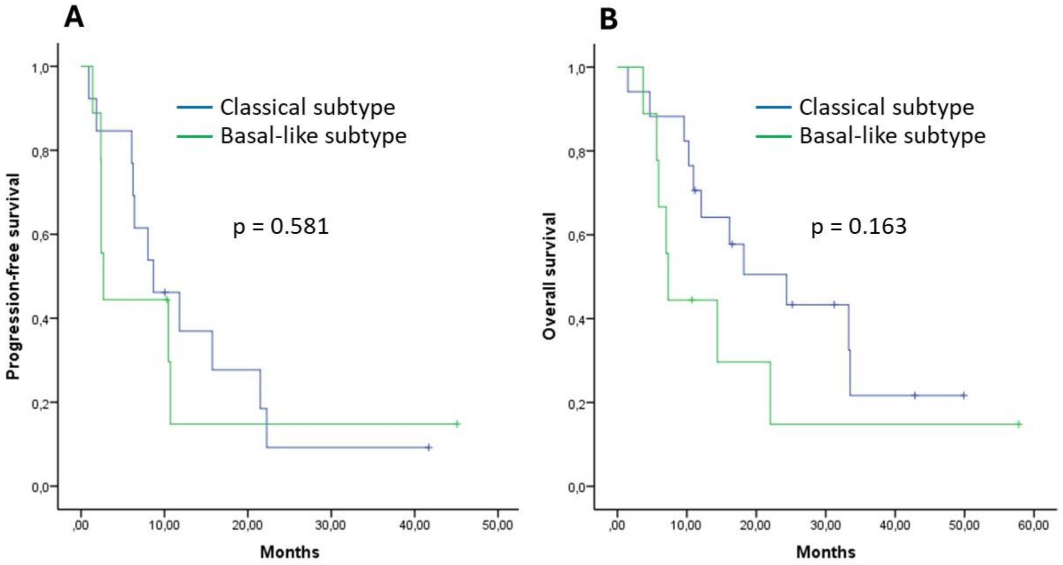

Supplementary Figure S3: Immunohistochemical characteristics of the classical subtype. GATA6 immunohistochemistry: nuclear staining intensity of 2, 50% positive tumor cells, and H-score of 100, image at 40x magnification (A). CK17 immunohistochemistry: membrane staining intensity of 0, 0% positive tumor cells, and H-score of 0, image at 40x magnification (B).

A B

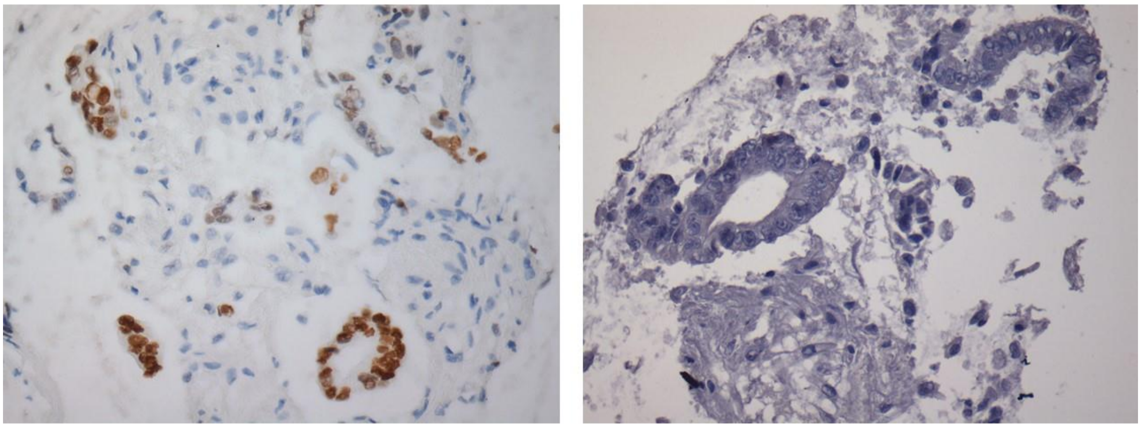

Supplementary Figure S4: Immunohistochemical characteristics of the basal-like subtype. GATA6 immunohistochemistry: nuclear staining intensity of 0, 0% positive tumor cells, and H-score of 0, image at 40x magnification (A). CK17 immunohistochemistry: membrane staining intensity of 1, 70% positive tumor cells, and H-score of 70, image at 40x magnification (B).

A B

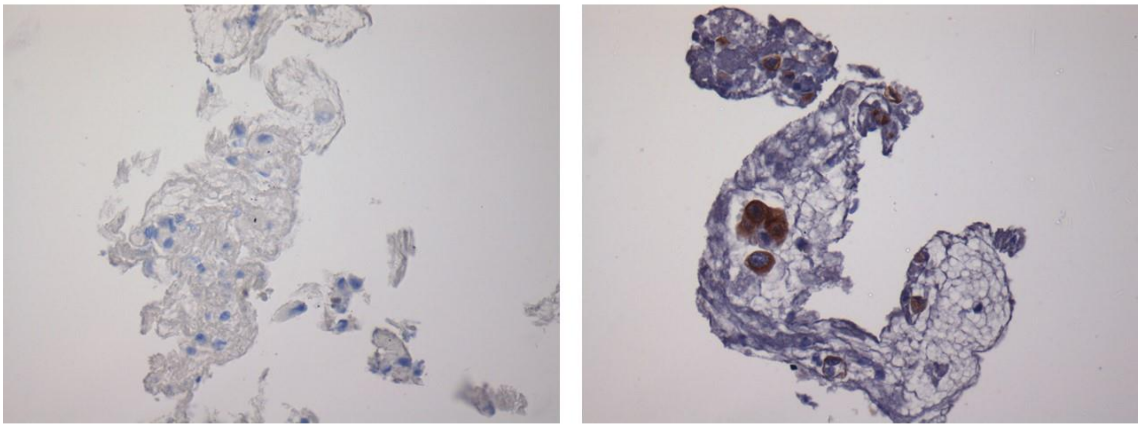

Supplementary Figure S5: Immunohistochemistry for the mixed subtype. GATA6 immunohistochemistry: nuclear staining Intensity of 3, 90% positive tumor cells, and H-Score of 270, image at 40x magnification (A). CK17 immunohistochemistry: membrane staining intensity of 2, 75% positive tumor cells, and H-Score of 150, image at 40x magnification (B).

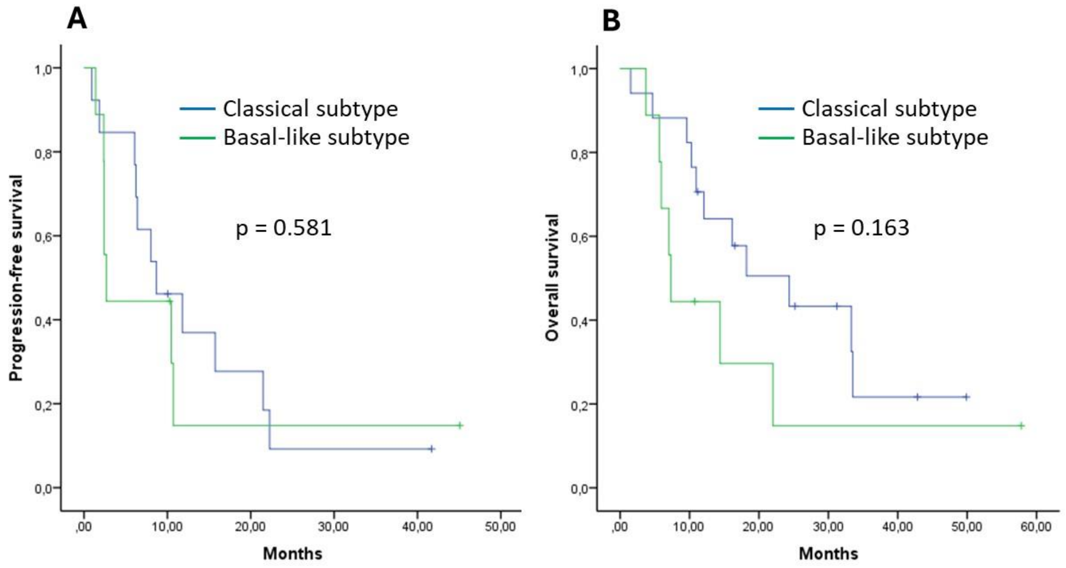
A
Progression-free survival
1,0
0,8
0,6
0,4
0,2
0,0
,00
10,00
20,00
30,00
40,00
50,00
Months
Classical subtype
Basal-like subtype
p = 0.581
B
Overall survival
1,0
0,8
0,6
0,4
0,2
0,0
,00
10,00
20,00
30,00
40,00
50,00
60,00
Months
Classical subtype
Basal-like subtype
p = 0.163
